# Application of oral inorganic iodine in the treatment of Graves’ disease

**DOI:** 10.3389/fendo.2023.1150036

**Published:** 2023-04-03

**Authors:** Yixuan Huang, Yihang Xu, Murong Xu, Xiaotong Zhao, Mingwei Chen

**Affiliations:** Department of Endocrinology, The First Affiliated Hospital of Anhui Medical University, Hefei, Anhui, China

**Keywords:** inorganic iodine, Graves’ disease, therapy, diet, special population

## Abstract

Iodine is a crucial trace element for the human body and the basic raw material for the synthesis of thyroid hormones. Oral inorganic iodine includes dietary iodine and therapeutic iodine, both of which are closely associated with thyroid immunity and metabolism. Graves’ disease (GD), also known as diffuse toxic goiter, is characterized by hyperthyroidism and high iodine metabolism. Clinically, patients diagnosed with GD are often asked to limit iodine intake or even avoid iodine in their diet. The latest research has demonstrated that the interference of dietary iodine with antithyroid drugs (ATDs) treatment may be overestimated. In addition, as a medication for GD treatment, the administration of inorganic iodine has shown positive results in patients with mild hyperthyroidism, a low thyroid autoantibody concentration, a small thyroid volume, a high iodine diet and so on. Inorganic iodine may also be used as an alternative when patients experience side effects with traditional ATDs and for those who still prefer conservative treatment. Due to its low teratogenicity, blood toxicity and bone marrow toxicity, inorganic iodine plays a unique role in special populations, such as pregnant or lactating patients and patients receiving tumor radiotherapy or chemotherapy. In this review, the research progress, biological function, doses and effects, applicable populations and specific applications of dietary iodine and therapeutic iodine are summarized to provide references for the diagnosis and treatment of GD, thus improving the quality of life of GD patients.

## 1 Introduction

The thyroid gland is an organ characterized by iodine enrichment, and the rate of iodine uptake is significantly increased in patients with Graves’ disease (GD). The role and importance of inorganic iodine in the pathogenesis, treatment and prevention of GD has always been an issue of concern (1). In daily life, the iodine needed to maintain the physiological state is realized through the absorption of iodine from consumed food and iodized salt. The recommended intake of iodine by the World Health Organization is 150 mcg per day for adults over 12 years of age and 250 mcg per day for pregnant and lactating women; the median urinary iodine concentration (UIC) should be controlled at 100-200 mcg per day to ensure that the population has an appropriate iodine nutritional status and is free from iodine deficiency or excess (2). In clinical practice, the oral administration of inorganic iodine is also used as a treatment for GD. Inorganic iodine drugs commonly used in the clinic include potassium iodide and sodium iodide. In China, Lugol’s solution is also approved for clinical use by the China Food and Drug Administration. Lugol’s solution is a composite iodine solution consisting of elemental iodine (5%), potassium iodide (10%) and distilled water. Since the solution tastes bitter and is irritating to the gastrointestinal tract, it is often recommended to fully dilute it with a sweet drink (3). In the clinic, inorganic iodine is generally used as an adjuvant in antithyroid drugs (ATDs); however, the effect of inorganic iodine alone on GD remains to be evaluated (4). In ATDs treatment, the oral administration of inorganic iodine may play an important role in the appearance of adverse reactions such as liver dysfunction, neutropenia and rash, yet patients may not meet the indications for surgery or radioiodine (RAI) therapy. In particular, the prospects for administration for women in the gestational and lactational periods, infants and young children, tumor patients and other special populations should be considered (5, 6). High-dose iodide results in the temporary suppression of thyroid hormone release and the escape of suppressed thyroid function, designated the Plummer effect and Wolff-Chaikoff effect, respectively (7, 8).Therefore, inorganic iodine is often administered in specific situations, such as in the preoperative stage and during thyroid storm (9), to stabilize patients’ metabolism and vital signs and to help subsequent treatment go smoothly. The applications and underlying related mechanisms remain to be further investigated and summarized to clarify the value and safety of inorganic iodine administration in patients with GD.

## 2 The mechanism of iodine in the thyroid gland

**Figure 1:**
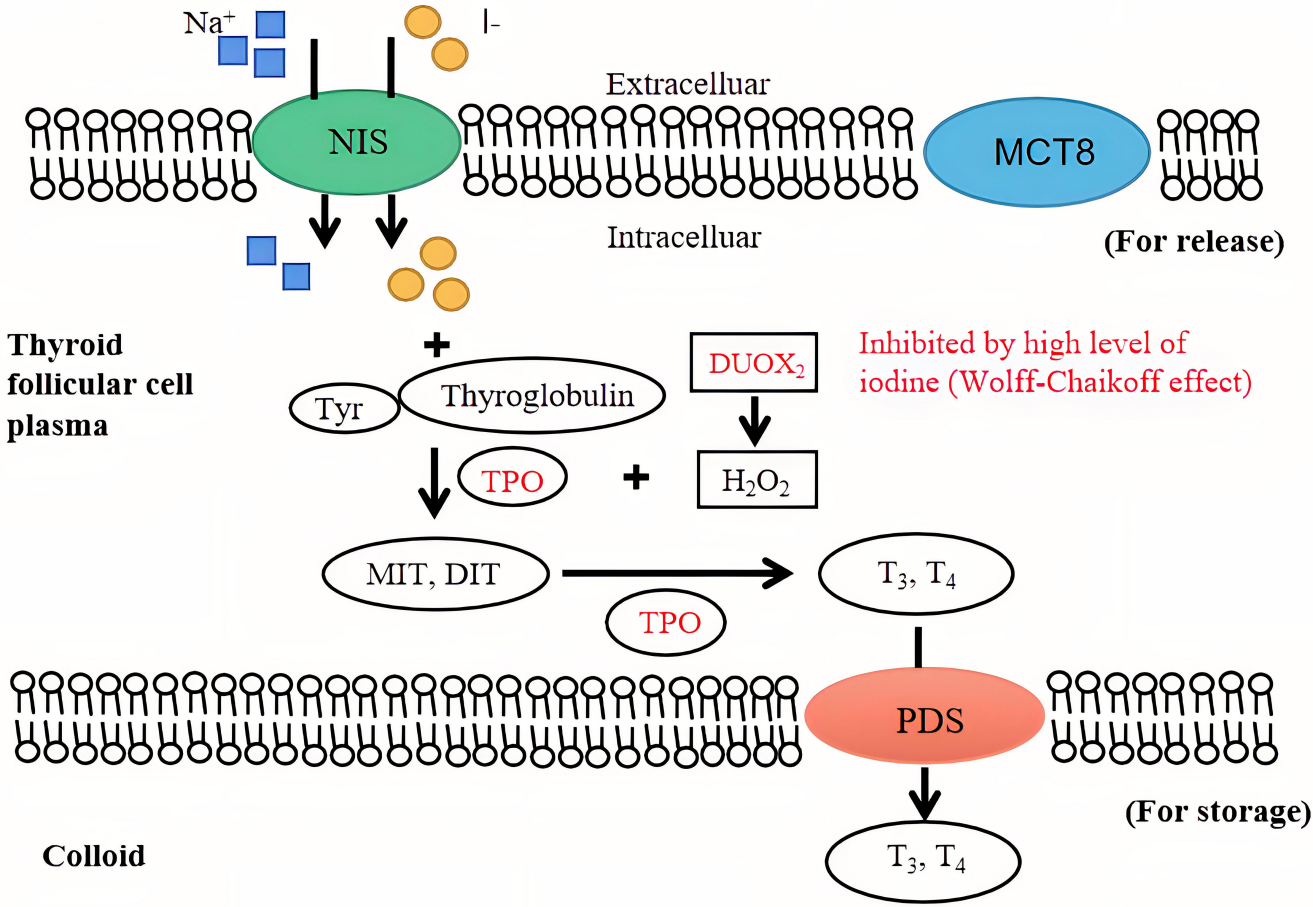
The mechanism of iodine in the thyroid gland. An intuitive view of the mechanism of normal and high iodine levels on the thyroid gland and thyroid hormone synthesis. The inhibition of high iodine levels on thyroid hormone synthesis (Wolff-Chaikoff effect) is shown in red. NIS, sodium iodide symporter; TPO, thyroid peroxidase; MIT, monoiodo-tyrosine; DIT, diiodo-tyrosine; T3, triiodothyronine; T4, thyroxine; PDS, anion exchanger pendrin; MCT8, monocarboxylate transporter 8.

The key molecular structure of iodine uptake in the thyroid gland is the sodium iodide symporter (NIS), which is located in the external basement membranes of thyroid follicular cells. The NIS couples the “uphill” inward transport of I− against its electrochemical gradient under the help of the “downhill” inward translocation of Na+ down its electrochemical gradient generated by the Na+/K+ ATPase (10). Thyroid peroxidase (TPO) utilizes hydrogen peroxide, which is mostly produced under the action of dual oxidase 2 (DUOX2) under physiological conditions, to combine iodine with the tyrosine residues of thyroglobulin molecules to form monoiodotyrosine and diiodotyrosine. Monoiodotyrosine and diiodotyrosine are coupled with TPO to form thyroid hormones, consisting of triiodothyronine (T3) and thyroxine (T4), most of which are transported to the thyroid colloid for storage by the anion exchanger pendrin (PDS) located at the apical membrane of follicular cells. Thyroid hormones are endocytosed through the thyroid colloid into the cytoplasm of thyroid follicular cells and transported to the blood through monocarboxylate transporter 8 (MCT8) when needed (11). An intuitive view of the mechanism of normal/high iodine levels in the thyroid gland is shown in **Figure 1**. It has been reported that the immune-related pathway enrichment map of nuclear erythroid factor 2 (Nrf2) knockout mice after exposure to iodide is 65.75%, consistent with that of GD mouse models. As a transcription factor, Nrf2 attenuates the inflammatory-autoimmune-fibrotic response activated by excessive iodide, which may be related to the pathological mechanism of GD (12). Recently, Eleftheriadou et al. demonstrated a higher positive rate of NIS autoantibodies in patients with thyroid disease than in healthy controls (7.7% vs. 1.8%) and an even higher positive rate in patients with GD (12.3%) (13). Supplementation with sodium perchlorate reduces the absorption of iodide by reducing the NIS transport rate of thyroid follicular cells in a concentration-dependent manner.

## 3 Dietary iodine and GD

### 3.1 Dietary iodine and the prevalence of GD

According to the latest global dietary iodine nutrition assessment released by the World Health Organization in 2020, the number of countries with sufficient iodine intake nearly doubled from 67 in 2003 to 118 in 2020 under the benefit of the implementation of the global salt iodization programme (14). A number of epidemiological surveys have shown that the prevalence of GD is higher in iodine-deficient areas (15). Wang et al. found that the proportion of GD in individuals with predominant and subclinical hyperthyroidism was different in areas with various iodine nutritional statuses in China and that the prevalence of GD reached the highest in the group with UICs less than 50 mcg/L. Multivariate regression analysis indicated that iodine deficiency was related to the prevalence of GD (OR = 1.67, 95% CI 1.30-2.15) (16). Subsequently, the gradual introduction of iodine replacement therapy to individuals in iodine-deficient areas greatly reduced the incidence of GD. For example, the risk of hyperthyroidism was halved in the Danish population through salt iodization (17). Li et al. conducted two repeated surveys on iodine intake and thyroid status in the Chinese population in 2009 and 2015. Comparing the survey results from 2009 and 2015, the prevalence rates of dominant hyperthyroidism, subclinical hyperthyroidism and GD were 0.7% and 0.5% (*P*<0.05), 0.5% and 0.3% (*P*<0.05), and 0.5% and 0.3% (*P*<0.01), respectively, leading to a decrease in the prevalence of hyperthyroidism and GD in the overall population (18). Notably, a compulsory universal salt iodization (CUSI)program was carried out in 1996 in China. Afterwards, the average iodine intake value in the Chinese population was excessive from 1996 to 2001, high-normal from 2002 to 2011, and intermediate from 2012 to 2016 (19). With these changes in iodine status, the prevalence of hyperthyroidism also changed. In the fourth year after the implementation of the CUSI program, the prevalence of GD was 1.25% (20), which decreased to 0.61% in the ninth year (21). At present, the CUSI program has been implemented for more than 20 years. The latest research shows that the current prevalence of GD in China is 0.53% (16). These results suggest that the effect of iodine status on hyperthyroidism is bidirectional. As iodine is an important raw material for the synthesis of thyroid hormones, deficient or excess iodine will both increase the risk of thyroid autoimmunity in adults. There is a U-shaped relationship between iodine status and thyroid autoimmunity risk in adults. The underlying mechanisms involve the activation of the immunogenicity of thyroid globulin, inducing an immune attack on the thyroid tissue itself (22). Selenium is also essential for thyroid hormone synthesis and function. Epidemiological studies have linked an increased risk of autoimmune thyroiditis, GD and goiter to low selenium status (23) and revealed that the cause of goiter after salt iodization may be related to the insufficient intake of selenium (24). There is evidence from observational studies and randomized controlled trials that selenium/selenoproteins reduce thyroid peroxidase antibody titres, hypothyroidism, and postpartum thyroiditis. Adequate selenium intake is vital in areas of iodine deficiency/excess. In areas with low selenium intake, it may be appropriate to supplement 50-100 micrograms of selenium every day (25), which can effectively reduce autoimmune thyroid disease. However, in a randomized double-blind, placebo-controlled, supplementary trial (26), no significant effect of selenium supplementation on GD remission and recurrence rates was found. In addition, although selenium supplementation can reduce circulating thyroid antibody titres, this change is also meaningless in the pathogenesis of GD (4). Therefore, the effect of selenium supplementation on GD pathogenesis and the efficacy of GD treatment needs further study.

### 3.2 Effect of dietary iodine on GD medications

Previous studies have shown evidence that GD patients in iodine-deficient areas have a higher recurrence rate after ATD treatment (27). In areas characterized by iodine deficiency, such as Sweden, Turkey and Ireland, the GD recurrence rate after ATD treatment was significantly higher than the average rate before the implementation of salt iodization policies in the last century, with recurrence rates ranging from 65% to 84% (28). The restriction of iodine intake increases the expression of thyroglobulin antibody (TgAb) and thyroid peroxidase antibody (TPOAb) under the regulation of negative feedback, resulting in a more serious autoimmune response (29). The findings of a multivariate regression analysis indicated that the increase in TPOAb had a great impact on the prevalence of GD. In contrast to TPOAb, only an increase in TgAb > 400IU/mL had a positive effect on the prevalence of GD (16). Huang et al. conducted a prospective study to compare the outcomes of ATD treatment under iodine supplementation and restriction conditions in patients with GD. Within 12 months after the cessation of ATDs, the recurrence rate in the iodine supplementation group was significantly lower than that in the iodine restriction group (35.5% vs. 45.5%) (30). Patients included in this study were all from high-iodine intake areas, and large amounts of thyroid hormones may have been stored in the thyroid gland in advance for those who were exposed to adequate iodine for a long period of time. In this case, a decrease in iodine intake may cause thyroid hormone regulation disorders. Therefore, it is unnecessary to restrict iodine intake to improve the effectiveness of ATDs and remission rate of GD in areas with sufficient or excessive iodine intake. To reduce the recurrence rate of GD following ATD treatment in iodine-deficient areas, an increase in the baseline iodine supply for the population is crucial. Therefore, in the evaluation of the long-term effect of ATDs on GD, attention should be given to the environmental iodine status.

## 4 Inorganic iodine as a drug in the treatment of GD

In terms of the effect of inorganic iodine as a therapeutic drug for GD, the administration of different doses should be considered. Potassium iodide is the most commonly used inorganic iodine reagent in the clinic. According to an observational study conducted by Okamura et al., 70.8% of the patients experienced remission when treated with less than 200 mg/d of potassium iodide, compared to 35.0% of the patients who experienced remission when treated with 200 mg/d or more (31). On the other hand, S. Nagataki et al. reported that all subjects experienced clinical improvement after treatment with potassium iodide at 30 mg/d; however, half of the patients experienced recurrence 4-16 weeks after treatment (32). Considering the limited amount of literature and the controversial conclusions, further research on the specific comparison between different doses and their potential effects is warranted. Among the potential effects, special attention should be given to the confusing effect of environmental iodine on the efficacy of inorganic iodine treatment.

Here, we evaluate the oral administration of potassium iodide in the treatment of GD among various populations, including general adults, pregnant and lactating women and patients with malignant tumors. The advantages and potential adverse effects of potassium iodide administration are also emphasized (**Table 1**).

**Table 1:** The administration of potassium iodine in different patients.

Subjects	Advantages	Potential adverse effects
General patients with GD	Achieve long-term remission in approximately 60% of mild GD patients (33), and prevent the need for a second radioiodine dose after the first RAI dose for GD (34).	Increased thyroid hormone (33) and thyroid autoantibody (35) levels; Escape of the antithyroid effect (5).
Special populations		
Pregnant GD patients	Reduce the risk of congenital malformations and adverse reactions to ATDs (5, 36)	Excessive iodine intake with potential risks (37, 38)
Lactating GD patients	Thyroid function is unaffected in most infants, and they are free from adverse reactions to ATDs (39)	Subclinical or dominant hypothyroidism in infants due to excessive iodine intake, especially in premature infants (40, 41)
GD patients complicated with malignant tumors	Reduce the risk of ATD-associated neutropenia in GD patients with malignant tumors, especially in elderly patients over 65 years old (42, 43)	Transient hypothyroidism in a few patients (43)

GD, Graves’ disease; ATDs, antithyroid drugs.

### 4.1 Oral administration of potassium iodide in the treatment of GD in general adults

Suzuki et al. conducted a 3-year prospective study on newly diagnosed patients with mild GD whose free tetraiodothyronine (fT4) levels were less than 5.0 ng/dL at the time of diagnosis. Patients were treated with an initial dose of potassium iodide of 50 mg/d, and if their fT4 values did not decrease to the upper limit of normal (1.6 ng/dL) after the initiation of treatment, the dose was increased to 100 mg/d. The results showed that 58.2% of the patients responded to potassium iodide monotherapy; that is, their fT4 level could be controlled in the normal range. When the fT4 level was <2.76 ng/dL, the effective rate of potassium iodide monotherapy was more than 79% (33). The findings of the study illustrated that potassium iodide has a certain curative effect and achieves a relatively high long-term remission rate in newly treated patients with mild GD. However, the conclusions currently apply only to areas with adequate iodine intake since the participants included in the study were characterized as having a high baseline iodine intake. Yoshihara et al. noted that the drug sensitivity of potassium iodide was less than 44% when the thyrotropin receptor antibody (TRAb) level was greater than 6.8 IU/L (5). GD patients with higher TRAb levels may have worse effects from potassium iodide treatment. Moreover, the findings of studies to date have shown the possibility of painless thyroiditis in GD patients treated with potassium iodide (35). It is worth noting that 90.9% of patients diagnosed with painless thyroiditis are TgAb- or TPOAb- positive. Potassium iodide induces autoimmune changes in patients. Therefore, seroconversion or a continuous increase in thyroid autoantibodies is a situation that should be considered in the treatment of GD with potassium iodide. In addition, a larger thyroid volume is associated with a higher risk of irresponsiveness to potassium iodide, so patients with increased thyroid volume during treatment tend to have a greater risk of recurrence (33).

In summary, the adverse factors affecting the efficacy of potassium iodide administration in the treatment of general adults with GD include a high thyroid hormone level, thyroid autoantibody concentration and thyroid volume. Potassium iodide administration is relatively safe for disease control in general adult patients with mild GD. Attention should be given to monitoring the changes in thyroid hormone levels and thyroid autoimmune indices during long-term treatment. The use of potassium iodide should be carefully terminated when the thyroid hormone level remains abnormal. Although iodine is effective in the treatment of hyperthyroidism in patients with mild GD, it is significantly less effective than ATDs in patients with more severe diseases. At present, based on the quality of evidence, the oral administration of potassium iodide is not recommended by authoritative guidelines in the treatment of GD in general adults.

### 4.2 Oral administration of potassium iodide in the treatment of GD in special populations

#### 4.2.1 Oral administration of potassium iodide in the treatment of gestational GD

Gestational GD has always been a focus in clinical treatment and often becomes tricky, especially with the appearance of ATD side effects. Although some clinical studies have shown that there is no correlation between exposure to antithyroid drugs (such as methimazole) in early pregnancy and birth defects (44) and antithyroid drugs are the first-line treatment in pregnant women with hyperthyroidism, other studies have shown that the incidence of neonatal congenital malformations increases after treatment with methimazole, and the incidence of maternal liver toxicity increases after treatment with propylthiouracil during the first trimester of pregnancy (36, 45). In addition, recent animal studies have revealed that ATDs interfere with hypothalamus-pituitary-thyroid axis signaling in a dose-dependent manner and induce the impairment of nervous system development in offspring (46). Therefore, potassium iodide treatment for GD has become an alternative. A retrospective study showed that the incidence of neonatal malformations in a group of patients who converted from methimazole to potassium iodide treatment in the first trimester [4/260 (1.53%)] was lower than that in the group treated with methimazole [47/1134 (4.14%)], and none of the neonates exposed to potassium iodide had thyroid dysfunction or goiter (36). The findings from another retrospective cohort study demonstrated that 55.4% of patients were in remission and successfully ceased taking drugs during pregnancy after changing from methimazole to potassium iodide therapy in the first trimester of pregnancy (5). Thus, potassium iodide treatment for GD in early pregnancy can effectively prevent the occurrence of neonatal congenital malformations, and a low dose of potassium iodide can be administered as an alternative therapy in pregnant patients with new-onset mild GD when side effects appear while taking ATDs. It should be noted that in many studies on the administration of potassium iodide for the treatment of GD during pregnancy, most GD patients used ATDs as the first-line treatment before switching to potassium iodide treatment, so the interference of ATDs on the observed outcomes of clinical treatment with potassium iodide cannot be ruled out. Therefore, further prospective studies are needed to evaluate the efficacy and foetal safety of inorganic iodine in the treatment of GD during pregnancy.

Animal experiments have confirmed the negative effects of excess iodine on brain development and hippocampal metabolism in offspring (37). Clinical studies have shown that there is strong evidence of a negative correlation between the peak neonatal TSH value and placental iodine level (r=0.763, P<0.001) (47). When pregnant women take excessive iodine, their foetuses are prone to hypothyroidism, which is disadvantageous to intrauterine growth and especially the development of the nervous system (38). Considering the individual differences in GD patients during pregnancy or the improper evaluation of their condition, the therapeutic iodine intake may be excessive, so close follow-up must be carried out after iodide treatment. Drug replacement or surgical treatment in the second trimester of pregnancy is recommended if hyperthyroidism is not well controlled in mothers. It should be particularly emphasized here that the studies investigated oral potassium iodide administration during pregnancy without regulating the daily dietary iodine intake. Although the dose of dietary iodine is much smaller, studies have shown that inadequate daily iodine intake increases the risk of hypothyroidism in healthy pregnant women (48). The effects of dietary iodine on drug iodine efficacy in pregnant women with GD need to be further studied. Although iodine in supraphysiological doses has been successfully used in the treatment of pregnant women with hyperthyroidism, the research data are mainly from the Japanese population. Therefore, the guidelines of the American Thyroid Association (ATA) and European Thyroid Association (ETA) recommend that additional data are needed before iodine therapy can be generally recommended for pregnant women with GD (49, 50).

#### 4.2.2 Oral administration of potassium iodide in the treatment of lactating GD patients

It is of great significance to clarify the effect of inorganic iodine on the thyroid function of lactating mothers with GD and their infants since numerous mothers prefer breastfeeding as much as possible. Recently, Hamada et al. evaluated the thyroid function of infants breastfed by mothers with GD who were treated with potassium iodide. The average dose of maternal potassium iodide was 50 mg/d. The results among 100 breast-fed infants demonstrated that 88 had normal thyroid hormone levels and 12 had subclinical hypothyroidism, but the blood TSH levels of all infants returned to normal within 2 months after the discontinuation of potassium iodide (39). Although the frequency of breastfeeding varies from infant to infant, there is no significant effect of frequency on the degree of iodine intake, and the concentration of iodine in urine is positively correlated with that in breast milk. Infants are not easily affected by excessive iodine, and the underlying cause might be immature self-regulation mechanisms, such as iodine inhibition, which are relatively weak compared with those of adults (51). Second, serum TRAb levels in pregnant women with GD often increase in the third trimester and pass through the placenta, which assists the thyroids of infants in resisting excess iodine until 2 to 3 months after birth (52, 53). Nonetheless, the monitoring of thyroid function in infants is highly recommended during inorganic iodine therapy for lactating mothers with GD. Breastfeeding should be stopped, and levothyroxine should be administered to infants if subclinical or dominant hypothyroidism is discovered. In addition, premature infants are characterized by poor metabolic function and vulnerability to excessive iodine. The breastfeeding of premature infants should be avoided by mothers receiving inorganic iodine treatment. There is still a lack of large sample assessment data on the extent to which premature infants are affected by iodine in breast milk (40, 41). Regarding the oral administration of potassium iodide in the treatment of lactating GD patients, it is impossible to assess the benefits or risks due to insufficient evidence. At present, the international authoritative guidelines do not provide corresponding recommendations.

#### 4.2.3 Oral administration of potassium iodide in the treatment of GD combined with malignant tumors in other organs

Neutropenia is an occasionally severe and potentially fatal adverse reaction to both chemotherapy and ATDs treatment (42, 54). Although the incidence is low, it may be difficult to differentiate whether neutropenia is chemotherapy-induced or ATDs-induced at the early stage of treatment in patients with GD combined with malignant tumors in other organs. Okamura et al. reported that approximately 1/3 of patients with untreated GD combined with malignant tumors in other organs were sensitive to potassium iodide therapy and achieved long-term remission, while approximately 1/3 of the patients experienced inorganic iodine escape. The inorganic iodine escape rate decreases to approximately 20% when the serum fT4 level is lower than 5 ng/dL, and the inorganic iodine escape rate decreases to approximately 10% when the patient is over 65 years old (43). The increase in the potassium iodide effect in elderly patients may be related to iodine accumulation and the decrease in renal iodine excretion function caused by age. GD patients showed varying degrees of sensitivity to potassium iodide, and nearly no side effects except hypothyroidism occurred. Therefore, it is worth attempting potassium iodide monotherapy when chemotherapy is needed in patients with both GD and malignant tumors and the underlying risk of bone marrow and blood toxicity is high, thus alleviating the possibility of neutropenia caused by thioamides or chemotherapeutic drugs.

## 5 The application of inorganic iodine in specific cases of GD

Recent studies have provided a diverse perspective on three special circumstances of potassium iodide application: GD patients undergoing thyroidectomy, GD patients receiving RAI treatment and GD patients experiencing thyroid storm (**Table 2**).

**Table 2:** The administration of potassium iodide under three special circumstances.

	Conventional understanding	Present viewpoint
Thyroidectomy in GD patients	High-dose potassium iodide can promote the rapid recovery of thyroid function, reduce intraoperative bleeding, and shorten the operation time.	Preoperative use of potassium iodide does not reduce the incidence of postoperative complications and the total length of hospital stay (55, 56). No solid evidence to support the necessity of preoperative iodine use.
RAI treatment in GD patients	Before RAI therapy, limiting iodine intake can improve its efficacy for GD.	It is not necessary to limit iodine intake before RAI treatment for GD (57). A restricted iodine diet only has a significant positive effect on the radiotherapy of thyroid cells (such as tumor cells) with a low iodine uptake rate (58).
Thyroid storm in GD patients	Short-term use of potassium iodide is beneficial to control hyperthyroidism storm through the Plummer effect.	In rare cases, an excess iodine load can aggravate thyrotoxicosis (59).

GD, Graves’ disease; RAI therapy, radioiodine therapy.

### 5.1 Potassium iodide and thyroidectomy in patients with GD

Potassium iodide is often used in clinical preparation for operations in GD patients based on the fact that large doses promote the rapid recovery of thyroid function and reduce intraoperative bleeding. Recent studies indicated that the incidence of postoperative complications was not reduced by the preoperative administration of potassium iodide, and there was no solid or conclusive evidence to support the necessity of preoperative iodine use (55, 56). No significant difference was shown in the probability of postoperative complications, such as recurrent laryngeal nerve injury, hypoparathyroidism or cervical haematoma, between patients who did and did not receive potassium iodide in clinical preparation. Although the operation time was significantly prolonged without the use of potassium iodide in clinical preparation, there was no impact on the total length of stay. Ali et al. performed a retrospective analysis, and the outcomes of urgent thyroidectomy following rapid control with potassium iodide were similar to those after elective operation with ATDs alone. No significant differences in complications, such as hypoparathyroidism, vocal cord paralysis or postoperative bleeding, were observed between the groups (60). Considering the difficulty of accurately ruling out confounding factors in retrospective analyses, multicenter double-blind randomized controlled trials are needed to further evaluate the effects of the preoperative administration of inorganic iodine. Despite the low quality of evidence, according to the international guidelines, short-duration and high-dose preoperative potassium iodide administration is still recommended for the following GD patients (43, 50, 61): (1) patients who are in need of urgent or elective thyroidectomy or other types of operations; (2) patients with side effects due to intolerability of ATDs or other types of drugs; (3) women in the second trimester of pregnancy; and (4) patients in whom anaesthesia duration must be controlled due to an underlying disease or state. The specific application method is that potassium iodide can be given as 5-7 drops (0.25-0.35 mL) of LS (8 mg iodide/drop) or 1-2 drops (0.05-0.1 mL) of a saturated solution (50 mg iodide/drop) mixed in water or juice three times daily for 10 days before surgery (49).

### 5.2 Potassium iodide and RAI treatment in patients with GD

NIS protein is expressed on the basolateral membrane of thyroid follicular cells, which is the common channel for RAI therapy and inorganic iodine intake. In the past, limited dietary iodine intake was always recommended for patients before RAI therapy and was especially suitable for patients with a relatively low radioactive iodine uptake rate to increase the proportion of trapped RAI. Iodine deficiency blocks thyroid hormone synthesis and increases TSH under feedback regulation, which stimulates an increase in NIS synthesis. However, a Japanese study conducted in 2021 reported that limiting iodine intake within 5 to 7 days before RAI therapy had no effect on GD treatment efficacy. No associations were observed between dietary iodine and therapeutic potassium iodide intake and the therapeutic effect of RAI (OR=0.974, 95% CI 0.956-0.993) (57). In addition, a clinical study from Germany confirmed that patients with GD taking 600 mg of inactive potassium iodide for three days during RAI therapy had significantly improved efficacy of RAI therapy and did not need a second RAI dose (34). Nevertheless, a restricted iodine diet has a significant positive effect on the radiotherapy of thyroid cells with a low iodine uptake rate, such as tumor cells (58), whereas NIS expression in thyroid cells is active and functional in the GD state, and the possibility of permanent hypothyroidism cannot be ignored if the iodine dose is not properly controlled. From this point of view, it is not necessary to impose strict limitations on dietary iodine and therapeutic inorganic iodine intake in patients with GD to obtain the maximum efficacy of RAI. However, the ATA pointed out that nutritional supplements that may contain excess iodine and seaweed should be avoided for at least 7 days (49).

### 5.3 Potassium iodide and thyroid storm in patients with GD

Thyroid storm, as an endocrine emergency characterized by severe thyrotoxicosis, can be secondary to poorly controlled GD. In the clinic, potassium iodide and other medications are often administered to control thyroid storm. The 2016 ATA guidelines recommended that a saturated potassium iodide solution be used. The specific usage is to take 5 drops (0.25 mL or 250 mg) orally every 6 hours (49). Nevertheless, a series of recently published case reports have shown that thyroid storm can likewise be induced by acute or excessive iodine load. A patient suddenly suffered from respiratory and circulatory failure and cardiac arrest after taking iodine-containing medications for six months after the diagnosis of GD. The serum and urinary iodine level was approximately 1000 times the normal value, and urgent haemodialysis was needed to eliminate excess iodine. In rare cases, the administration of exogenous iodine induces hyperthyroidism in patients with abnormal thyroid function, known as Jod-Basedow syndrome (59). Dhami reported the case of a patient who was diagnosed with GD one year prior and experienced sudden cardiac arrest due to myocardial infarction caused by an iodine-containing contrast agent used during percutaneous coronary intervention (62). Transient coronary vasospasm, ischaemic ventricular fibrillation and cardiac arrest were caused by the injection of an iodine contrast agent, aggravating the symptoms and manifestations of myocardial infarction. Therefore, iodine contrast agents should be used cautiously in patients with GD complicated with cardiovascular disease. The contradictory phenomenon of the mechanisms of thyroid autoregulation and abnormal regulation during excess iodine intake is described as the Wolff-Chaikoff effect and Jod-Basedow syndrome, respectively. Jod-Basedow syndrome is a manifestation of the evasion of the physiological negative feedback mechanism of the Wolf-Chaikoff effect. The change in autoimmune mechanisms may be involved in the transition between the two effects (63).

## 6 Conclusions

Inorganic iodine is closely related to thyroid autoimmunity, oxidative stress and inflammation. It is of great value to explore the application prospects of inorganic iodine for patients with GD, whether as a substance in the daily diet or as a therapeutic medication. No sufficient evidence has been revealed to support the necessity of the strict limitation or compulsory use of iodine in GD patients during ATD treatment, before RAI treatment or before elective thyroidectomy. The efficacy of inorganic iodine as an oral medication in the treatment of GD is affected by thyroid function, thyroid volume and baseline iodine intake. Inorganic iodine also shows a certain degree of effectiveness and safety for special GD patients, such as those who are pregnant, lactating, have malignant tumors in other organs or are undergoing radiotherapy and chemotherapy. At present, based on the quality of evidence, the use of iodine-containing drugs in the preoperative period for hyperthyroidism and thyroid storm patients is strongly recommended by multinational guidelines. The evidence for the use of iodine-containing drugs for hyperthyroidism in other clinical settings needs to be further substantiated. It should be noted that the efficacy of inorganic iodine in the treatment of GD is closely related to environmental iodine, which needs to be considered in clinical applications. There are two sides to every coin. Inorganic iodine not only treats but also induces thyroid storm. More clinical and basic research studies are needed to verify the indications and contraindications.

## Author contributions

YH, YX, MX, and XZ performed the literature search and data analysis. YH and YX wrote the first draft of the manuscript, and all authors commented on the previous versions of the manuscript. MC drafted and critically revised the work. All authors contributed to the article and approved the submitted version.
